# On the Efficacy of Olive Oil as a Purgative, after the Ineffectual Trials of More Active Remedies. Also, an Account of an Intermittent, Cured by Nasal Hæmorrhage; and of a Chronic Intermittent Suspended by a Scald

**Published:** 1808-01

**Authors:** John Vaughan


### *On the Efficacy of Olive Oil as a Purgative, after the ineffectual Trials of more active Remedies. Also, an Account of an Intermittent, cured by nasal Hæmorrhage; and of a chronic Intermittent suspended by a Scald.* By Dr. John Vaughan.

Sir,

If you deem the following cases worthy of a place in the Medical Journal, they may, probably, not be uninteresting to your readers. Though theories entertain and instruct, we are often, in practice, disappointed in the operation of medicines. Peculiar states of system, frequently preclude the use of remedies which would otherwise promise the most success.

Mr. ———, aged 50, has, for ten years, been subject to occasional attacks of the cholic, with obstinate constipation of the bowels. On the 15th ult. I was called to him, and found him excessively distressed with flatulency and tormina, and frequent vomiting. He had, previously, taken two ounces of sulphat of soda, an ounce of castor oil, and a considerable quantity of manna and senna, without effect. His pulse being rather full, and apprehending inflammation, I drew a few ounces of blood, and commenced the use of purgatives and active injections. In the course of the following night, a dark coloured matter, of

 a susa suspicious appearance, was discharged by vomit, and my patient expressed himself sensible of inverted peristaltic motion, commencing low in the left hypogastrium, which excited apprehension of introsusception or iliac passion. The alarming condition of my patient, produced the most unremitted diligence in his attendants. Cathartics of different kinds and in varied forms were assiduously given, and enemas of salts and senna—of assafœtida—of carminative teas with ol. ricini—of tobacco, &c. were frequently exhibited, and fomentations applied to the abdomen, with but little effect. A respite from the more urgent symptoms was, however, obtained for two days, when the tormina and vomiting returned. Medicine seemed only to irritate the primæ viæ and increase his distress; and, while pondering on the use of quicksilver, it occurred to me that, perhaps, olive oil, from its bland taste and aperient quality, might not offend the stomach, and, if given in frequent doses, might eventually glide through the intestines. Accordingly, half an ounce of sallad oil was given every hour, alternated with injections of fennel-seed tea and castor oil. In six hours considerable relief was obtained, and several discharges procured. For two days my patient appeared convalescent, but incautiously taking three of Fothergill's pills, as *a simple laxative* (though composed of colocynth, aloes, scammony, and calx of antimony) he suffered a return of tormina and vomiting; but two ounces of olive oil, at one dose, and two or three injections, effectually cured. The oil passed through the bowels unmixed with feculent matter, and he was speedily restored to health.

It is, at the first view, somewhat irrational to suppose that so mild an aperient as olive oil should prove cathartic, when the routine of active purgatives have failed; but it was suggested by the circumstances already mentioned, and its utility was proved by the event. I should certainly have given the quicksilver, had it not been for the unpleasant reflections of the relatives, in case of an unfavourable event. Though no consideration should counteract the use of remedies, offering even a probability of success in desperate cases; yet if the object can be attained without aggravating the distresses of relatives, already great enough, the more agreeable alternative should be chosen.

If quicksilver operate only from its weight, it is an inoffensive remedy, in case it pass the bowels; but the same weight, which would overcome serious obstruction in the intestinal intestinal canal, might, in case of insuperable resistance, force its way through the coats of the bowels. Whether the olive oil would prove equally successful in a second case or not, is difficult to determine; and though the use of any article, in a single case, is not to be relied on as a general remedy, yet it is worthy of future trial.

### *Case of Intermittent Fever cured by Nasal Hæmorrhage.*

Mrs. ———, while labouring under an obstinate intermittent fever, in the seventh month of pregnancy, was attacked with nasal hæmorrhage, which resisted all the usual remedies, and was finally stopped by sponge forced up the nostril, so as to press on the mouth of the ruptured vessel. In five days, she was supposed to have lost four pounds of blood, which effectually cured her previous disease.

Blood-letting is often useful in intermittent fever, but it was the excessive discharge in this case, inducing a different diseased action, which banished the febrile disorder.

### *Case of Chronic Intermittent, suspended by a Scald of the Foot.*

A girl, who had had a chronic periodical fever for several weeks, accidentally scalded her foot, which remained sore for two weeks, and during this period she was freed from fever; but, when the ulcer healed, her fever returned. It is not improbable, but if the ulcer had been kept open a fortnight longer, or an epispastic added, in this case the cure would have been completed.

Wilmington, *February* 8, 1805.

